# Gastrectomy in comprehensive treatment of advanced gastric cancer with synchronous liver metastasis: a prospectively comparative study

**DOI:** 10.1186/s12957-015-0627-1

**Published:** 2015-07-01

**Authors:** Ziyu Li, Biao Fan, Fei Shan, Lei Tang, Zhaode Bu, Aiwen Wu, Lianhai Zhang, Xiaojiang Wu, Xianglong Zong, Shuangxi Li, Hui Ren, Jiafu Ji

**Affiliations:** Key Laboratory of Carcinogenesis and Translational Research (Ministry of Education), Department of Gastrointestinal Surgery, Peking University Cancer Hospital & Institute, 52 Fu-Cheng Road, Hai-Dian District, Beijing 100142 China; Key Laboratory of Carcinogenesis and Translational Research (Ministry of Education), Department of Radiology, Peking University Cancer Hospital & Institute, Beijing, China

**Keywords:** Advanced gastric cancer, Liver metastasis, Adjuvant gastrectomy, Hepatic resection

## Abstract

**Background:**

Systemic chemotherapy is the key treatment for advanced gastric cancer. The benefit of adjuvant surgery following preoperative chemotherapy in gastric cancer with liver metastasis has not been well established.

**Methods:**

Forty-nine gastric cancer patients diagnosed with synchronous liver metastasis initially treated with chemotherapy were categorized into the following two groups: surgery group: 25 patients who underwent gastrectomy and subsequently received postoperative chemotherapy and control group: 24 patients who received chemotherapy alone.

**Results:**

The median overall survival of patients in the surgery group and control group was 20.5 and 9.1 months, respectively, (*P* = 0.006). The median progression-free survival in the surgery group was 10.9 months, with statistical significance when compared with 5.0 months in the control group (*P* = 0.001). Multivariate analysis demonstrated that response to chemotherapy was the only independent factor in predicting prognosis. The survival of patients who achieved partial response (PR) was prolonged if they received adjuvant surgery (*P* = 0.024). No significant difference in the survival of patients underwent combined hepatic resection when compared with patients performed gastrectomy only.

**Conclusions:**

For gastric cancer with synchronous liver metastasis, adjuvant gastrectomy followed by chemotherapy might be beneficial for survival comparing with chemotherapy alone, especially in patients response to initial preoperative chemotherapy.

**Electronic supplementary material:**

The online version of this article (doi:10.1186/s12957-015-0627-1) contains supplementary material, which is available to authorized users.

## Background

Gastric cancer is one of the most common malignancies in the world. In China, it is the second leading cancer cause of death ranking after lung cancer [1]. It is commonly detected at advanced stages for its initial stage often lack of specific symptom. Liver metastasis is one of the prognostic factors predicting poor survival in gastric cancer. Nearly 5–10 % of gastric cancer patients have involvement of synchronous liver metastasis when get diagnosis [2].

Systemic chemotherapy is the key treatment for advanced gastric cancer (AGC). Multiple trails of various chemotherapeutic regimens have been conducted to prolong the survival of patients with AGC. Classical chemotherapy regimens for AGC include CF and ECF [3]. Paclitaxel is an anti-mitotic drug which had been widely used in the treatment of a variety of tumors including gastric cancer. Clinical trials have confirmed the safety and efficiency of paclitaxel and capecitabine (PX) combination chemotherapy in AGC [4–6].

Although great progress has been achieved in chemotherapy, the outcome of patients with AGC is still poor. Tumor progression is common in patients with temporary regression after chemotherapy due to drug resistance. Surgery is another way to get a long-time survival in the patient with advanced cancer. The significance of surgery for AGC, especially for the liver metastasis from gastric cancer, is still elusive [7–14]. Recently, Suzuki et al. reported that adjuvant surgery was effective following the response of chemotherapy in AGC patients with liver metastasis [15]. In some cases, the adjuvant surgery resulted in further long-term survival. Nevertheless, solid evidence from prospective study is lacking.

This study therefore aimed to investigate whether adjuvant surgery followed by chemotherapy was more beneficial than chemotherapy alone in gastric cancer patients with synchronous liver metastasis following preoperative chemotherapy.

## Methods

### Patients and study scheme

This study included 49 patients with AGC treated between June 2008 and December 2011 in Beijing Cancer Hospital. All patients were diagnosed with synchronous liver metastases and placed on a chemotherapy regimen, paclitaxel plus capecitabine (PX). Then, patients were divided into two groups based on their preference after multidisciplinary team (MDT) discussion. The surgery group underwent adjuvant gastrectomy (D2) followed by postoperative chemotherapy (PX). The control group received the same chemotherapy as described above without any operation (Additional file 1). Informed written consent was obtained from all patients, and the study was approved by the ethical committee in Beijing Cancer Hospital, China.

### Inclusion criteria

Patients were histologically proven gastric adenocarcinoma with hepatic metastasis identified by CT/MRI (AJCC TNM Version 7). No peritoneal or other distant metastasis; age range, ≥18 years; Karnofsky score ≥70; D2 lymph node dissection at palliative gastrectomy (no. of lymph node ≥15); no concurrent diseases that can cause death in 3 months; adequate major organ function (hemoglobin ≥90 g/L; neutrophil count ≥1.5 × 10^9^/L; platelet count ≥100 × 10^9^/L; ALB ≥30 g/L; AST, ALT, and ALP ≤2.5 times the upper limit of normal; total bilirubin <1.5 times the upper limit of normal; creatinine lower than the upper limit of normal).

### Chemotherapy regimen

Capecitabine (1000 mg/m^2^) was administered orally within 30 min after morning and evening meals for 2 weeks, followed by a drug-free interval of 1 week (one cycle). Paclitaxel (80 mg/m^2^) was diluted in 0.9 % saline and administered as a 2-h infusion in the morning of day 1 and day 8 of each cycle (i.e., every 3 weeks). The paclitaxel infusion was started simultaneously with the capecitabine administration. Responses were classified according to the RECIST guidelines. Patients in the surgery group were placed on postoperative chemotherapy within 4 to 6 weeks after gastrectomy.

### Definition of adjuvant gastrectomy

Patients with progressive disease are considered not suitable for resection. Adjuvant gastric resection included the absence of primary tumor, both macroscopically and microscopically, by D2 lymphadenectomy. Resection margin was not less than 5 cm for partial gastrectomy. In total gastrectomy, proximal resection margin was not less than 2 cm while distal resection margin was not less than 5 cm. Simultaneous hepatic resection was conducted when the liver metastasis could be curative resected through the evaluation.

### Follow-up

Patients were assessed every month to detect any adverse events with verbal interview, physical examination, and blood tests, including a complete blood cell count and measurements of liver and renal function, until disease progression. Abdominal CT/MRI and measurements of CEA and CA19-9 were carried out every 3 months. Overall survival was defined as the time from diagnosis of AGC with synchronous liver metastases to death from any cause or last follow-up. Progression-free survival (PFS) was defined as the length of time after treatment during which the disease did not get worse.

### Statistical analysis

Data related to patient characteristics were compared between the two groups by using the chi-square test. Data on patients who were alive or lost to follow-up were censored. The primary end point was survival. Cumulative survival was estimated with the Kaplan-Meier method (SPSS version 19 software), and comparisons between the groups were done with a log-rank test. A multivariate analysis of the Cox proportional hazards regression model (backward, stepwise) was created to assess the influence of each variable on survival. Significance was set at *P* < 0.05.

## Results

### Patient characteristics

A total of 49 AGC patients with synchronous liver metastases were treated in this study. Table 1 shows the patient characteristics. There were no major imbalances between the two groups in terms of characteristics of primary gastric cancer except with regard to the primary tumor location. In the surgery group, 25 % of the primary tumor occurred at EGJ or upper third of stomach, significantly lower than 42.9 % of which in the control group (*P* = 0.003). No statistical significance was identified in age, Borrmann type, histological grade, pathological classification, and T stage between the two groups (*P* > 0.05).

**Table 1:** Clinical pathological data of AGC patients with synchronous liver metastasis

Clinical pathological data	Group underwent adjuvant gastrectomy	Group treated with chemotherapy only	*P* value
Age	61.4 ± 9.5	60.8 ± 7.9	0.834
BSA	1.758 ± 0.186	1.743 ± 0.142	0.755
Gender (male vs. female)	3.2:1	5:1	0.725
Primary tumor location			0.003
EGJ	4 (16.7 %)	0 (0 %)	
U	2 (8.3 %)	9 (42.9 %)	
M	3 (12.5 %)	5 (23.8 %)	
L	15 (62.5 %)	7 (33.3 %)	
Borrmann type			0.355
I	0 (0 %)	0 (0 %)	
II	3 (13.6 %)	4 (21.1 %)	
III	19 (86.4 %)	14 (73.7 %)	
IV	0 (0 %)	1 (5.3 %)	
Histological grade			0.594
Low differentiation	9 (40.9 %)	9 (45.0 %)	
Median and low differentiation	0 (0 %)	1 (5.0 %)	
Median differentiation	4 (18.2 %)	4 (20.0 %)	
High and median differentiation	9 (40.9 %)	6 (30.0 %)	
Pathological classification			0.273
Adenocarcinoma	22 (91.7 %)	21 (100 %)	
Small cell carcinoma	1 (4.2 %)	0 (0 %)	
Signet ring cell carcinoma	1 (4.2 %)	0 (0 %)	
T stage (TNM version 7)			0.387
T2	3 (13.0 %)	1 (5.0 %)	
T3	1 (4.3 %)	0 (0 %)	
T4a	17 (73.9 %)	15 (75.0 %)	
T4b	2 (8.7 %)	4 (20.0 %)	

### Treatment

All 49 patients were placed on three courses of chemotherapy (PX). Response evaluation of patients after chemotherapy was shown in Additional file 2. In the surgery group, 14 (60.9 %) patients achieved partial response (PR) and 8 (34.8 %) patients reached stable disease (SD), significantly higher when compared with 7 (31.8 %) PR and 3 (13.6 %) SD in the control group (*P* = 0.001). All patients were assessed for toxicities that are listed in Additional file 3. Patients were generally well tolerated throughout the study. Adverse events associated with PX were observed in 31 (63.3 %) patients. The most common adverse effects were fatigue (44.9 %) and anemia (36.7 %). Grade 4 adverse events were rare. Dose reduction in chemotherapy occurred in five (12.2 %) patients.

A total of 25 patients underwent adjuvant gastrectomy followed by postoperative chemotherapy. As shown in Additional file 4, eight (32.0 %) patients received total gastrectomy. Thirteen (52 %) patients underwent combined hepatic resection with gastrectomy. Postoperative complications included gastroparesis and abdominal infections occurred in four (16 %) patients.

### Patient survival

**Fig. 1:**
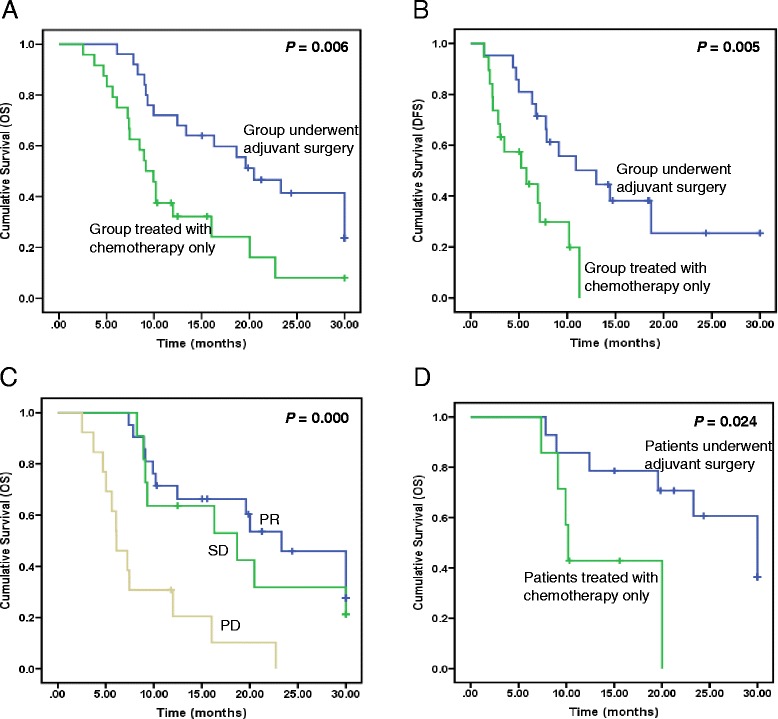
Kaplan-Meier curves for survival in AGC patients with liver metastasis. **a**, **b** Comparison of OS and PFS between the adjuvant surgery and chemotherapy group. **c** Comparison of OS among the various responses to chemotherapy (PR, SD, and PD). **d** Comparison of OS between patients who initially achieved PR after chemotherapy then underwent adjuvant surgery and patients who only treated with chemotherapy initially achieved PR

Median lengths of follow-up in the surgery group and control group were 19.6 and 9.5 months, respectively. The median overall survival (OS) of patients in the surgery group was 20.5 months, which was statistically more prolonged than 9.1 months in the control group (Fig. 1a, *P* = 0.006). The 1- and 2-year survival rates were 72 and 32 % in the surgery group and 41 and 8 % in the control group, respectively. In addition, the median PFS in the surgery group was 13.0 months, with statistical significance when compared with 5.8 months in the control group (Fig. 1b, *P* = 0.005).

We next analyzed the survival efficacy of chemotherapy in all AGC patients. Patients were divided into three groups based on the response to PX. The median OS of patients who achieved PR was 23.3 months, significantly longer than 18.7 and 6.1 months in patients who achieved SD and progression disease (PD), respectively (Fig. 1c, *P* < 0.001). In this study, more patients who achieved PR and SD were enrolled in the surgery group than in the control group after MDT discussion. Subsequently, we performed the subgroup analysis according to the response to chemotherapy. The median OS in patients who achieved PR after chemotherapy in the surgery and control groups was 30.0 and 10.2 months, respectively, with significant statistical difference (Fig. 1d, *P* = 0.024). No significant difference of OS was identified in patients who reached SD after chemotherapy (data not shown).

**Fig. 2:**
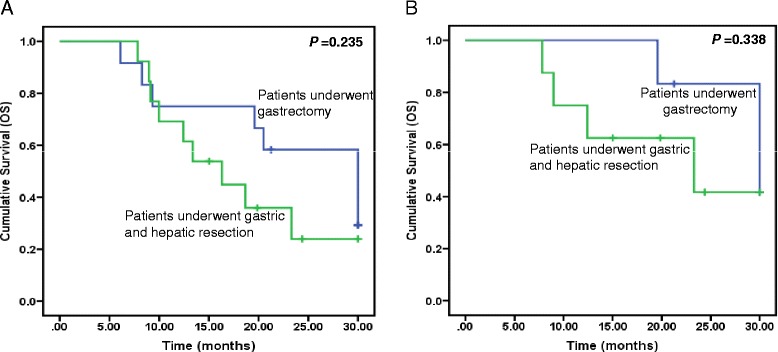
Kaplan-Meier curves for survival in patients who underwent adjuvant surgery. **a** Comparison of OS between patients who underwent gastrectomy and patients treated with combined gastric and hepatic resection. **b** Comparison of OS between patients who initially achieved PR after chemotherapy then underwent gastrectomy and patients who initially achieved PR after chemotherapy then treated with combined gastric and hepatic resection

To illustrate the efficacy of the hepatic resection in AGC with liver metastasis, we compared the survival of patients in the surgery group who underwent combined hepatic resection or not. The median OS was 16.3 months in patients with combined hepatic resection, with no statistical significance when compared with 30.0 months in patients without hepatic resection (Fig. 2a, *P* = 0.235). Importantly, among the patients who have achieved PR after preoperative chemotherapy, the median OS in patients who underwent combined gastric and hepatic resection was 23.3 months, with no statistical significance when compared with 30.0 months in patients treated with gastrectomy (Fig. 2b, *P* = 0.338).

**Fig. 3:**
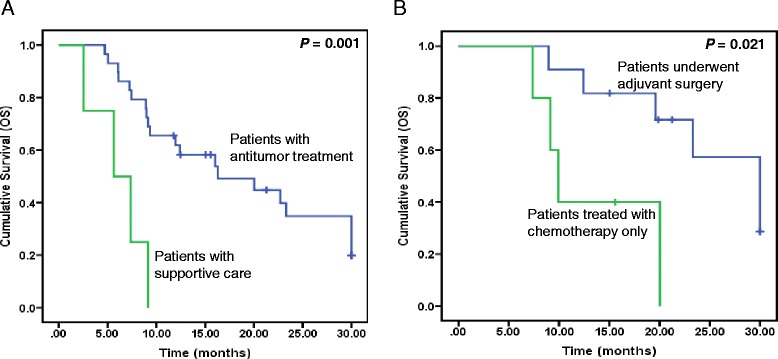
Kaplan-Meier curves for survival in patients with tumor progression. **a** Comparison of OS between patients with anti-tumor treatment and supportive care after tumor progression. **b** Comparison of OS between the adjuvant surgery and chemotherapy group among patients with tumor progression who initially reached PR after chemotherapy

Among all 49 patients, during the 2-year follow-up, 43 patients developed imaging-defined progression. Then, we classified the patients into two groups: one underwent anti-tumor treatment after progression and the other received supportive care after progression. The median OS was 16.3 months in the group underwent anti-tumor treatment, with statistical significance when compared with 5.6 months in the group received supportive care after progression (Fig. 3a, *P* = 0.001). Among the 43 patients, 16 patients have reached PR after initial chemotherapy. Eleven of the 16 patients underwent adjuvant gastrectomy, while the remaining patients were only treated with chemotherapy. The median OS of the 11 patients was 30.0 months, significantly prolonged than 9.9 months in the remaining ones (Fig. 3b, *P* = 0.021).

### Univariate and multivariate analyses of prognosis factors

The results of the univariate analysis are shown in Additional file 5. The following two factors were found to be univariately related to better outcomes: response to chemotherapy (*P* < 0.001) and adjuvant gastrectomy (*P* = 0.006). No survival difference was found in age, gender, primary tumor location, Bormann type, pathological classification, and T stage (*P* > 0.05). Multivariate analysis demonstrated response to chemotherapy as an independent factor in predicting prognosis (Table 2, *P* = 0.026).

**Table 2:** Multivariate analysis of prognostic factors for OS of AGC patients with synchronous liver metastasis

Variables	HR	CI (95 %)	*P* value
Underwent gastrectomy	1.327	0.501–3.517	0.569
Response evaluation			0.026
SD vs. PR	0.228	0.076–0.682	0.008
PD vs. PR	0.292	0.096–0.888	0.030

## Discussion

Gastric cancer is the leading cause of cancer-related death in the world. Outcome for patients with AGC is extremely poor. The treatment strategy for AGC with synchronous liver metastasis is still not well established. Combined chemotherapy is a way to improve survival and quality of life for patients with AGC. Kang et al. conducted a phase II study of PX to evaluate this combination in AGC [6]. Results were promising which included high response rate (44.5 % PRs) and prolonged patient survival (median OS: 11.3 months). In our current, single-center trial of AGC with synchronous liver metastasis, consistent with previous studies, pretreatment with PX resulted in an overall response rate of 42.8 % (PR).

Referring the efficacy of surgery for AGC, gastrectomy is indicated to provide palliation of the active symptoms such as bleeding and obstruction; nevertheless, the role of gastric resection in the treatment of patients with minimal symptoms and non-curative factors including liver metastasis remains controversial. Studies have reported improved survival in AGC patients who underwent palliative gastrectomy when compared to those who had not [9, 10, 12]. Lin et al. reported that both survival time and palliative duration were significantly longer in patients with stage IV gastric cancer after palliative gastrectomy than non-resection operations [12]; however, others implied that no patients or only a selected subsets of patients with AGC might benefit from non-curative gastrectomy [8, 11]. Li et al. demonstrated that only patients with single peritoneal dissemination had survival benefit from palliative resection while others with single liver, distant lymph nodes, or multiple sites metastasis had none [11]. One possible reason for the persistent controversy is that heterogeneous patients with different clinical parameters were enrolled in different studies. For example, multiorgan metastasis could be a factor for predicting poor prognosis of AGC.

Recently, increasing studies have evaluated the combined efficacy of non-curative surgery with peri-operative systemic chemotherapy in AGCs. The preoperative chemotherapy aimed to downstage the primary tumor while postoperative chemotherapy was administered to treat the residual cancer and micrometastasis. Kokkola et al. reported that patients with metastatic gastric cancer underwent gastrectomy followed by postoperative chemotherapy had better median OS than patients who underwent gastrectomy without chemotherapy [16]. In another study, the median OS of AGC patients underwent gastrectomies after the response to chemotherapy reached 28.5 months [15], which was more prolonged than the range for median survival (5~24 months) in patients who underwent non-curative gastrectomy [17]. Patients might benefit from the non-curative surgery when preoperative chemotherapy was effective. However, most of these studies were retrospective studies with limited number of patients enrolled, and prospective trials are warranted to determine the value of adjuvant gastrectomy following preoperative chemotherapy in AGCs.

In this study, we prospectively compared the efficacy of adjuvant surgery plus chemotherapy (PX) with chemotherapy alone following the response to neoadjuvant/preoperative chemotherapy for AGC with synchronous liver metastasis. Both median OS and PFS of patients in the surgery group were statistically more prolonged than chemotherapy-only group. However, in the multivariate analysis, response to chemotherapy was the only positive prognostic factor in AGCs, with no effect on the adjuvant surgery. In this trial, more patients who achieved PR and SD were enrolled in the surgery group than in the control group for a curative intention. Poor survival in the control group might be caused by high PD rate in this group. In the subgroup analyses, the survival of patients who achieved PR was prolonged if they received adjuvant surgery. For this reason, a subgroup of patient could benefit from adjuvant surgery following the response to chemotherapy.

Another important issue is about the surgical resection of hepatic metastasis from AGC. Liver metastases from gastric cancer are rarely recommended to surgery for they are often complicated with other distant metastasis. A number of studies have reported that the efficacy of hepatic resection for liver metastases from gastric cancer was doubtful, while others showed that the combined hepatic resection leads to long-term survival in some selected patients [7, 13, 14]. Even though, indications for surgery could be considered relay on the analysis of prognostic factors for gastric cancer with liver metastasis. Patients with good prognostic capacity might benefit from hepatic resection. Kodera et al. concluded that both “number of metastatic nodules” and “solitary tumor” were common independent prognostic factors for the gastric cancer with hepatic metastasis [18]. Others indicated that patients with synchronous solitary liver metastasis and without serosal invasion could be the criteria for liver resection [19, 20]. To enrich the evidence for combined hepatic resection in AGC with liver metastasis, we compared the survival of patients who underwent liver surgery or not. Results showed that patients who underwent gastrectomy plus hepatic resection suffered poor outcomes. No clinical benefit of hepatic resection was found when AGC patients with liver metastasis were treated. The possible reason is that most of the patients enrolled in our study were diagnosed with multiple metastasis nodules. Systemic therapy is still the main strategy for patients with multiple metastasis lesions.

In addition, treatment failure is common in AGC patients with liver metastasis. The optimal treatment strategy after treatment failure remains a clinical concern. In our study, most of the patients who have failed the initial treatment accepted a secondary anti-tumor therapy including systemic chemotherapy, radiotherapy, or interventional treatment. The remaining four patients who received supportive care all died in 10 months. The anti-tumor treatment prolonged the survival to 16.3 months. Thus, patients who failed the initial treatment would benefit from secondary anti-tumor therapy. Interestingly, among patients with treatment failure, 16 of them reached PR after chemotherapy. Eleven of the 16 patients who underwent gastrectomy had better outcome than the rest who did not treat with surgery. Accordingly, patients with tumor progression following a temporary regression to chemotherapy could also benefit from the adjuvant surgery after the initial chemotherapy.

## Conclusions

PX is a promising combined chemotherapy regimen for AGC with liver metastasis. Response to chemotherapy is the only independent prognostic factor for patient survival. Adjuvant gastrectomy is reasonable in patients response to preoperative chemotherapy. More evidence is needed for the resection of liver metastasis from AGC.

## Additional files

Additional file 1:
**The study scheme.** Patients were enrolled into two groups based on their preference after MDT discussion. Additional file 2:
**Response evaluation of all AGC patients with synchronous liver metastasis after three courses of chemotherapy (PX).** More patients in the surgery group achieved PR and SD after chemotherapy than in control group. Additional file 3:
**Adverse events of chemotherapy in all 49 patients.** Patients were generally well tolerated in this study. Additional file 4:
**Summary of 25 AGC patients underwent adjuvant gastrectomy.** About half patients underwent combined hepatic resection with gastrectomy. Additional file 5:
**Univariate analysis of prognostic factors for OS of AGC patients with synchronous liver metastasis.** Response to chemotherapy and adjuvant gastrectomy were factors related to better outcomes.

## Abbreviations

AGC: advanced gastric cancer
CA19-9: carbohydrate antigen 19–9
CEA: carcinoembryonic antigen
CF: cisplatin and fluorouracil
ECF: epirubicin, cisplatin, and fluorouracil
EGJ: esophagogastric junction
MDT: multidisciplinary team
OS: overall survival
PD: progression disease
PFS: progression-free survival
PR: partial response
PX: paclitaxel and capecitabine
SD: stable disease
