# Supplementary figures and images for: Gastrectomy in comprehensive treatment of advanced gastric cancer with synchronous liver metastasis: a prospectively comparative study
Source: World J Surg Oncol. 2015 Jul 1;13:212. doi: 10.1186/s12957-015-0627-1 (PMC4491213; doi:10.1186/s12957-015-0627-1)

**Additional file 1.**  
The study scheme

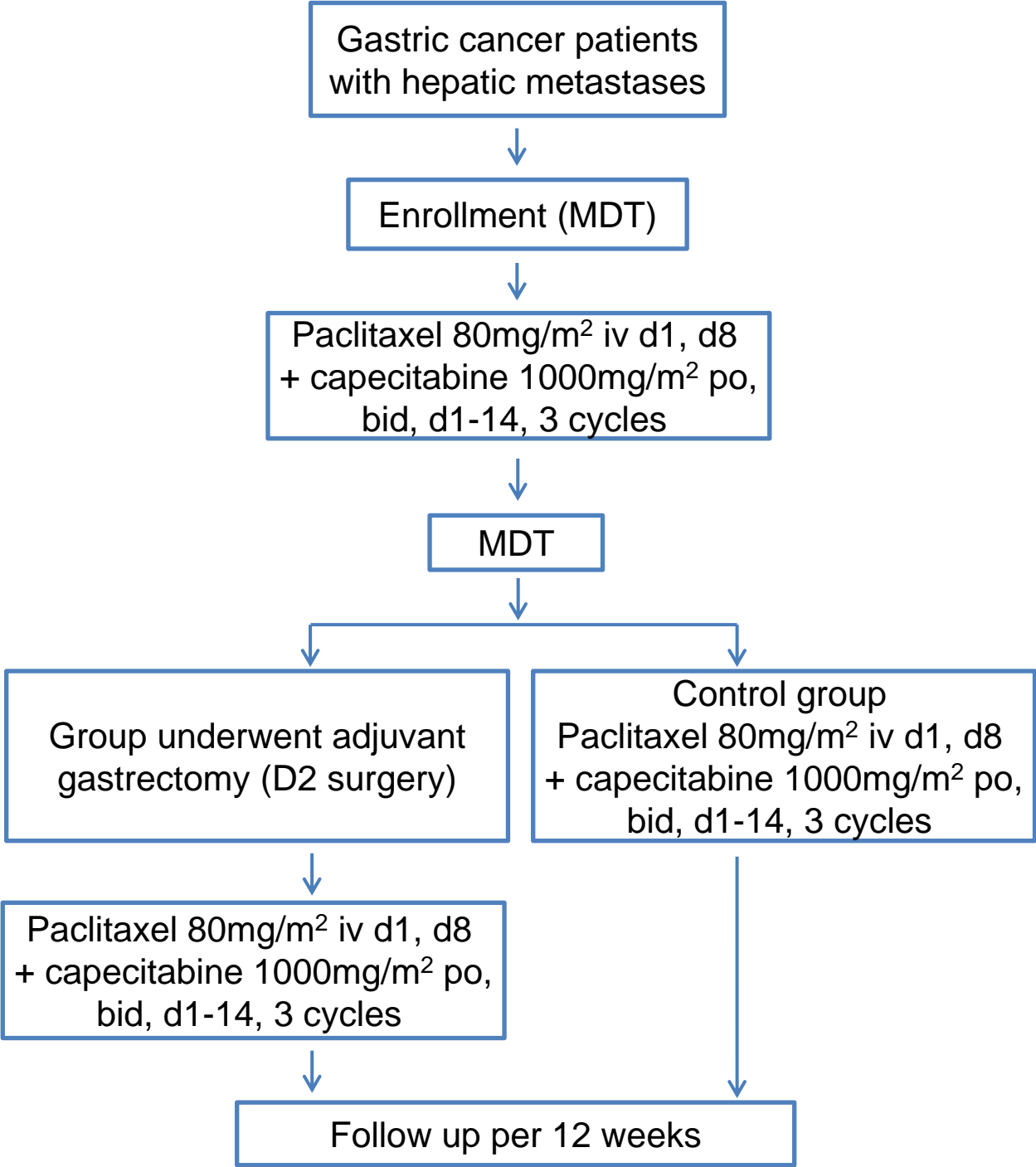

Supplement: Additional file 1: — The study scheme. Patients were enrolled into two groups based on their preference after MDT discussion. [file 12957_2015_627_MOESM1_ESM.pdf]
