# Supplementary material for: Gastrectomy in comprehensive treatment of advanced gastric cancer with synchronous liver metastasis: a prospectively comparative study
Source: World J Surg Oncol. 2015 Jul 1;13:212. doi: 10.1186/s12957-015-0627-1 (PMC4491213; doi:10.1186/s12957-015-0627-1)
Supplement: Additional file 2: — Response evaluation of all AGC patients with synchronous liver metastasis after three courses of chemotherapy (PX). More patients in the surgery group achieved PR and SD after chemotherapy than in control group. [file 12957_2015_627_MOESM2_ESM.pdf]

Additional file 2. Response evaluation of all AGC patients with synchronous liver metastasis after three courses of chemotherapy (PX).

	Group underwent adjuvant gastrectomy	Group treated with chemotherapy only	*P* value
Response evaluation			0.001
PR	14（60.9%）	7（31.8%）	
SD	8（34.8%）	3（13.6%）	
PD	1（4.3%）	12（54.4%）	

Additional file 3. Adverse events of chemotherapy in all 49 patients.

	No. of patients (%)				
AE	Grade 1	Grade 2	Grade 3	Grade 4	Grade 1-4
Leukopenia	-	2（4.1%）	2（4.1%）	-	4（8.2%）
Neutropenia	1（2.0%）	2（4.1%）	3（6.1%）	-	6（12.2%）
Anemia	11（22.4%）	6（12.2%）	1（2.0%）	-	18（36.7%）
Nausea	8（16.3%）	-	2（4.1%）	-	10（20.4%）
Vomiting	4（8.2%）	2（4.1%）	2（4.1%）	-	8（16.3%）
Fatigue	22（44.9%）	-	-	-	22（44.9%）
Decreased appetite	13（26.5%）	-	1（2.0%）	-	14（28.6%）
Diarrhea	3（6.1%）	-	1（2.0%）	-	4（8.2%）
Peripheral sensory neuropathy	7（14.3%）	-	-	-	7（14.3%）
Edema	2（4.1%）	-	-	-	2（4.1%）
Alopecia	1（2.0%）	2（4.1%）	-	-	3（6.1%）
Hyperpigmentation	2（4.1%）	-	-	-	2（4.1%）
Elevated ALT values	1（2.0%）	-	-	-	1（2.0%）
Hyperbilirubinemia	1（2.0%）	-	-	-	1（2.0%）
Mucositis	3（6.1%）	1（2.0%）		-	4（8.2%）
Palmar-plantar erythrodysesthesia syndrome	5（10.2%）	-	1（2.0%）	-	6（12.2%）
Pain	-	-	1（2.0%）	-	1（2.0%）

Additional file 4. Summary of 25 AGC patients underwent adjuvant gastrectomy.

Items	Patients underwent adjuvant gastrectomy
Type of gastrectomy	
Proximal subtotal gastrectomy	3（12.0%）
Distal subtotal gastrectomy	14（56.0%）
Total gastrectomy	8（32.0%）
Combined hepatic resection for liver metastases	
Yes	13（52%）
No	12（48%）
Digestive tract reconstruction	
Residual stomach esophagus anastomosis	3（12.0%）
Billroth I	10（40.0%）
Billroth II	2（8.0%）
Roux-en-Y	5（20.0%）
Jejunal pounch interposition reconstruction	5（20.0%）
Postoperative complication	4（16.0%）
Gastroparesis	2（8%）
Abdominal infection	2（8%）
No. of total lymph nodes	26.4±10.2
No. of lymph nodes with metastasis	6.6±5.0
N stage	
N0	3（12.0%）
N1	3（12.0%）
N2	5（20.0%）
N3	11（44.0%）

Additional file 5. Univariate analysis of prognostic factors for OS of AGC patients with synchronous liver metastasis.

Variables	Median OS (months)	*P* value
Age		0.784
< = 70	12.4（5.3-19.6）	
> 70	16.0（1.1-31.0）	
Gender		0.089
Male	16.0（6.7-25.4）	
Female	9.1（6.1-12.2）	
Response evaluation		<0.001
PR	23.3（15.6-30.9）	
SD	18.7（5.2-32.1）	
PD	6.1（4.2-8.0）	
Underwent gastrectomy		0.006
Yes	20.5（13.9-27.1）	
No	9.1（7.5-10.8）	
Primary tumor location		0.170
EGJ	16.3（5.3-27.3）	
U	9.1（7.5-10.8）	
M	19.6（0-42.3）	
L	18.7（6.0-31.3）	
Borrmann type		0.517
I	0	
II	12.9	
III	14.6	
IV	12.0	
Pathological classification		0.095
Adenocarcinoma	16.3（6.8-25.8）	
Small cell carcinoma	10.0	
Signet ring cell carcinoma	6.1	
T stage（TNM version 7）		0.351
T2	12.4	
T3	18.7	
T4a	9.1（7.8-10.5）	
T4b	16.0（7.4-24.7）	
