# Supplementary material for: Gastrectomy in comprehensive treatment of advanced gastric cancer with synchronous liver metastasis: a prospectively comparative study
Source: World J Surg Oncol. 2015 Jul 1;13:212. doi: 10.1186/s12957-015-0627-1 (PMC4491213; doi:10.1186/s12957-015-0627-1)
Supplement: Additional file 3: — Adverse events of chemotherapy in all 49 patients. Patients were generally well tolerated in this study. [file 12957_2015_627_MOESM3_ESM.pdf]

**Additional file 3**. Adverse events of chemotherapy in all 49 patients.

| | No. of patients (%) | | | | |
|---|---|---|---|---|---|
| AE | Grade 1 | Grade 2 | Grade 3 | Grade 4 | Grade 1-4 |
| Leukopenia | - | 2（4.1%） | 2（4.1%） | - | 4（8.2%） |
| Neutropenia | 1（2.0%） | 2（4.1%） | 3（6.1%） | - | 6（12.2%） |
| Anemia | 11（22.4%） | 6（12.2%） | 1（2.0%） | - | 18（36.7%） |
| Nausea | 8（16.3%） | - | 2（4.1%） | - | 10（20.4%） |
| Vomiting | 4（8.2%） | 2（4.1%） | 2（4.1%） | - | 8（16.3%） |
| Fatigue | 22（44.9%） | - | - | - | 22（44.9%） |
| Decreased appetite | 13（26.5%） | - | 1（2.0%） | - | 14（28.6%） |
| Diarrhea | 3（6.1%） | - | 1（2.0%） | - | 4（8.2%） |
| Peripheral sensory neuropathy | 7（14.3%） | - | - | - | 7（14.3%） |
| Edema | 2（4.1%） | - | - | - | 2（4.1%） |
| Alopecia | 1（2.0%） | 2（4.1%） | - | - | 3（6.1%） |
| Hyperpigmentation | 2（4.1%） | - | - | - | 2（4.1%） |
| Elevated ALT values | 1（2.0%） | - | - | - | 1（2.0%） |
| Hyperbilirubinemia | 1（2.0%） | - | - | - | 1（2.0%） |
| Mucositis | 3（6.1%） | 1（2.0%） | | - | 4（8.2%） |
| Palmar-plantar erythrodysesthesia syndrome | 5（10.2%） | - | 1（2.0%） | - | 6（12.2%） |
| Pain | - | - | 1（2.0%） | - | 1（2.0%） |
