# Supplementary material for: Gastrectomy in comprehensive treatment of advanced gastric cancer with synchronous liver metastasis: a prospectively comparative study
Source: World J Surg Oncol. 2015 Jul 1;13:212. doi: 10.1186/s12957-015-0627-1 (PMC4491213; doi:10.1186/s12957-015-0627-1)
Supplement: Additional file 4: — Summary of 25 AGC patients underwent adjuvant gastrectomy. About half patients underwent combined hepatic resection with gastrectomy. [file 12957_2015_627_MOESM4_ESM.pdf]

Additional file 4. Summary of 25 AGC patients underwent adjuvant gastrectomy.

Items	Patients underwent adjuvant gastrectomy
Type of gastrectomy	
Proximal subtotal gastrectomy	3（12.0%）
Distal subtotal gastrectomy	14（56.0%）
Total gastrectomy	8（32.0%）
Combined hepatic resection for liver metastases	
Yes	13（52%）
No	12（48%）
Digestive tract reconstruction	
Residual stomach esophagus anastomosis	3（12.0%）
Billroth I	10（40.0%）
Billroth II	2（8.0%）
Roux-en-Y	5（20.0%）
Jejunal pounch interposition reconstruction	5（20.0%）
Postoperative complication	4（16.0%）
Gastroparesis	2（8%）
Abdominal infection	2（8%）
No. of total lymph nodes	26.4±10.2
No. of lymph nodes with metastasis	6.6±5.0
N stage	
N0	3（12.0%）
N1	3（12.0%）
N2	5（20.0%）
N3	11（44.0%）
