# Supplementary material for: Gastrectomy in comprehensive treatment of advanced gastric cancer with synchronous liver metastasis: a prospectively comparative study
Source: World J Surg Oncol. 2015 Jul 1;13:212. doi: 10.1186/s12957-015-0627-1 (PMC4491213; doi:10.1186/s12957-015-0627-1)
Supplement: Additional file 5: — Univariate analysis of prognostic factors for OS of AGC patients with synchronous liver metastasis. Response to chemotherapy and adjuvant gastrectomy were factors related to better outcomes. [file 12957_2015_627_MOESM5_ESM.pdf]

Additional file 5. Univariate analysis of prognostic factors for OS of AGC patients with synchronous liver metastasis.

Variables	Median OS (months)	*P* value
Age		0.784
< = 70	12.4（5.3-19.6）	
> 70	16.0（1.1-31.0）	
Gender		0.089
Male	16.0（6.7-25.4）	
Female	9.1（6.1-12.2）	
Response evaluation		<0.001
PR	23.3（15.6-30.9）	
SD	18.7（5.2-32.1）	
PD	6.1（4.2-8.0）	
Underwent gastrectomy		0.006
Yes	20.5（13.9-27.1）	
No	9.1（7.5-10.8）	
Primary tumor location		0.170
EGJ	16.3（5.3-27.3）	
U	9.1（7.5-10.8）	
M	19.6（0-42.3）	
L	18.7（6.0-31.3）	
Borrmann type		0.517
I	0	
II	12.9	
III	14.6	
IV	12.0	
Pathological classification		0.095
Adenocarcinoma	16.3（6.8-25.8）	
Small cell carcinoma	10.0	
Signet ring cell carcinoma	6.1	
T stage（TNM version 7）		0.351
T2	12.4	
T3	18.7	
T4a	9.1（7.8-10.5）	
T4b	16.0（7.4-24.7）	
